# Modeling of Recommendation System Based on Emotional Information and Collaborative Filtering

**DOI:** 10.3390/s21061997

**Published:** 2021-03-12

**Authors:** Tae-Yeun Kim, Hoon Ko, Sung-Hwan Kim, Ho-Da Kim

**Affiliations:** 1 National Program of Excellence in Software Center, Chosun University, Gwangju 61452, Korea; tykim@chosun.ac.kr (T.-Y.K.); shkimtop@chosun.ac.kr (S.-H.K.); 2 IT Research Institute, Chosun University, Gwangju 61452, Korea; skoh21@chosun.ac.kr

**Keywords:** collaborative filtering, emotion recognition, support vector machine algorithm, speech emotion information

## Abstract

Emotion information represents a user’s current emotional state and can be used in a variety of applications, such as cultural content services that recommend music according to user emotional states and user emotion monitoring. To increase user satisfaction, recommendation methods must understand and reflect user characteristics and circumstances, such as individual preferences and emotions. However, most recommendation methods do not reflect such characteristics accurately and are unable to increase user satisfaction. In this paper, six human emotions (neutral, happy, sad, angry, surprised, and bored) are broadly defined to consider user speech emotion information and recommend matching content. The “genetic algorithms as a feature selection method” (GAFS) algorithm was used to classify normalized speech according to speech emotion information. We used a support vector machine (SVM) algorithm and selected an optimal kernel function for recognizing the six target emotions. Performance evaluation results for each kernel function revealed that the radial basis function (RBF) kernel function yielded the highest emotion recognition accuracy of 86.98%. Additionally, content data (images and music) were classified based on emotion information using factor analysis, correspondence analysis, and Euclidean distance. Finally, speech information that was classified based on emotions and emotion information that was recognized through a collaborative filtering technique were used to predict user emotional preferences and recommend content that matched user emotions in a mobile application.

## 1. Introduction

Emotion information and communication technology (ICT) is quickly becoming a core technology in the fields of smart mobile technology and wearable technology [1]. Emotion ICT automatically recognizes user emotions and processes information according to user environments to provide services that are emotionally customized [2]. Emotion signal sensing technology refers to ultra-small/ultra-precise sensor element technology that can sense biological signals, environmental/circumstantial information, image signals, speech signals, etc., based on autonomic nervous system activities triggered by changes in human emotions in an unrestrained/unconscious manner during daily life. Emotion detection technology processes and analyzes the signals acquired by sensors and recognizes, verifies, and standardizes human emotions based on these signals to digitize emotions [3]. Additionally, emotion service technology processes information according to user circumstances and provides emotionally customized products and services [4,5].

Human emotions are expressed in a variety of forms. Speech converts text-based information into sound that contains both emotions and lexical meaning. Factors such as the use of onomatopoeia, vocalization speed, and the length of pauses between phonations are useful clues for detecting a subject’s emotions [6]. Speech is the most efficient and natural type of human–machine interfacing and research on extracting the emotions contained in speech is very active. The results of previous speech recognition studies can serve as a starting point for speech-based emotion recognition. However, previous studies vary widely in terms of their selection of feature extraction and pattern recognition algorithms. Regarding the selection of feature vectors, speech recognition methods mainly use elements that model phonemes, whereas emotion recognition uses prosody elements. In addition to feature selection, the selection of pattern recognition algorithms is an important aspect. Different pattern recognition algorithms may be selected according to the methods used for modeling emotions based on extracted features [7,8]. Emotion information represents a user’s current emotional state and can be used in a variety of applications, such as cultural content services that recommend music according to user emotional states and user emotion monitoring [9].

Research on recommendation techniques that consider user tendencies to incorporate various user requirements effectively is also underway. Application programs that include recommendation techniques are used to predict items that will interest users and recommend those items [10,11]. A typical recommendation technique is content-based collaborative filtering. Content-based recommendation techniques directly analyze content to examine the similarities between content items and between content items and user preferences. New content is then recommended based on the results of this analysis. Collaborative filtering analyzes users who have tendencies that are similar to those of other users and estimates their content preferences [12,13]. To increase user satisfaction, recommendation techniques must understand and reflect user characteristics and circumstances, such as individual preferences and emotions. However, most recommendation techniques do not consider these characteristics and are unable to increase user satisfaction.

Emotion recognition is a technology that identifies the emotional state by analyzing information related to speech and gestures. The gestures can vary according to the culture. In adults, emotion-related information extracted through the speech is more consistent than that using facial expressions such as gestures, because adults tend to control their emotions. The objective of speech emotion recognition (SER) is to extract features from speech signals and then define, learn, and classify emotion models [14]. For emotion modeling, the hidden Markov model (HMM) was mainly used in the past. However, the recent emergence of deep neural networks (DNNs) and recurrent neural networks (RNNs) has enabled remarkable progress in research on recognition systems of time-series data, such as speech signals [15]. The study by Issa et al. introduced an architecture that extracted mel-frequency cepstral coefficients (MFCC), chromagram, mel-scale spectrogram, Tonnetz representation, and spectral contrast features from speech files and used them as inputs for one-dimensional convolutional neural networks (CNN). In addition, an incremental method that employed samples from the datasets of the Ryerson Audio-Visual Database of Emotional Speech and Song (RAV-DESS), Berlin (EMO-DB), and Interactive Emotional Dyadic Motion Capture (IEMOCAP) to modify the initial model for improving emotion recognition and classification accuracy was used [16]. The study by Sajjad et al. proposed a framework for speech emotion recognition (SER) using key sequence segment selection based on the redial-based function network (RBFN) similarity measure. The selected sequence was converted into a spectrum program by applying the short-time Fourier transform (STFT) algorithm and transferred to a CNN model to extract distinctive and prominent features from the speech spectrum program. By normalizing the CNN function and supplying it to a bi-directional long short-term memory (BiLSTM), time information for recognizing the final emotional state was learned [17]. The study by Wang et al. proposed a bimodal fusion algorithm for realizing voice emotion recognition by a weighted decision fusion method for facial expressions and voice information. This algorithm achieved facial emotion recognition by combining CNN and long short-term memory (LATM) RNN, and then transformed the speech signal into an image using MFCC [18]. These studies used frame unit features, pronunciation unit features, and a combination of LSTM RNN, DNN, and the simple concentration structure model and conducted a performance evaluation using datasets such as EMO-DB or IEMOCAP.

With the recent rapid developments in deep learning, excellent performance has been achieved after actual implementation, and many studies on artificial intelligence (AI) are being actively conducted. However, selecting the feature vectors of speech signals that express emotions well is as important as selecting the accurate classification engine in a speech-signal-based emotion recognition system. These systems show a lower recognition rate than other emotion recognition systems, such as facial expression recognition, not because of the low performance of the system itself but due to the inefficient extraction and selection of speech features.

Therefore, the present study aims to find an efficient and appropriate feature vector set for emotion classification with the goal of improving the performance of the emotion recognition system using speech signals, and it expects to achieve a higher emotion recognition rate. The speech data used in this study are from a Korean-style emotion speech database appropriate for Korean language and culture. The emotions were categorized into the six categories of normal, joy, sadness, anger, surprise, and boredom. A total of 2400 files with 400 data for each emotion were used as data for this recognition system, consisting of an equivalent proportion of male and female speech. An SVM classifier was used as the classification algorithm in this study. For image emotion information, 20 color emotion models were selected as the representative elements. Factor and correspondence analyses were conducted using a five-point-scale questionnaire survey, and the emotional spaces for each color were generated and measured. Furthermore, for music emotion information, the Euclidean distance was used to recommend the appropriate music for the current emotion according to the speech emotion information of the user based on their emotion history. Thus, using the property of emotional information, i.e., the preferred item varying according to the emotion of the user, we attempted to propose a system that recommends different content according to the emotion of the user. This was conducted by merging collaboration filtering with static emotional information that was received in real time from users. We also attempted to improve the performance through experiments.

## 2. Configuration and Design

The proposed content recommendation system, which uses speech emotion information and collaborative filtering, mainly consists of an emotion classification module, emotion collaborative filtering module, and mobile application.

**Figure 1:**
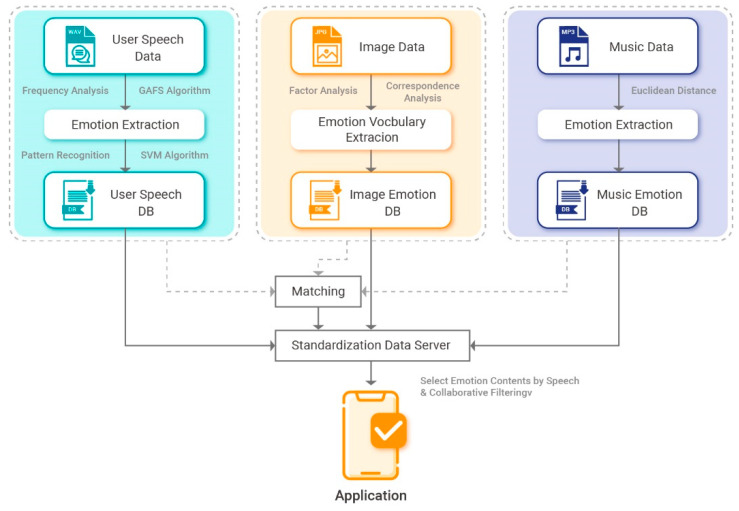
System configuration.

The structure of the proposed system is presented in Figure 1.

### 2.1. Emotion Classification Module

#### 2.1.1. Emotion Model Selection

In the field of emotion recognition, a systematic emotion model must be established to predict emotional states accurately. Human emotions are varied and complex and can be expressed by a large number of adjectives. Studies on quantifying emotional states and examining the correlations between emotional states are ongoing. Two emotion models that are often used in the field of emotion recognition are the Russel model and Thayer’s valence–arousal model. The former represents human emotions in a two-dimensional space with a positive–negative preference axis and an active–passive axis. The valence–arousal model is an emotion model that is often used in emotion recognition studies and depicts various emotions in a different two-dimensional space. The Russell model is an adjective-based model that has a disadvantage in that it uses overlapping meanings and ambiguous adjectival expressions [19]. The Thayer model overcomes these disadvantages by defining various emotional states using a valence axis that represents tendencies regarding emotions and an arousal axis that represents the strength of emotions [20].

**Figure 2:**
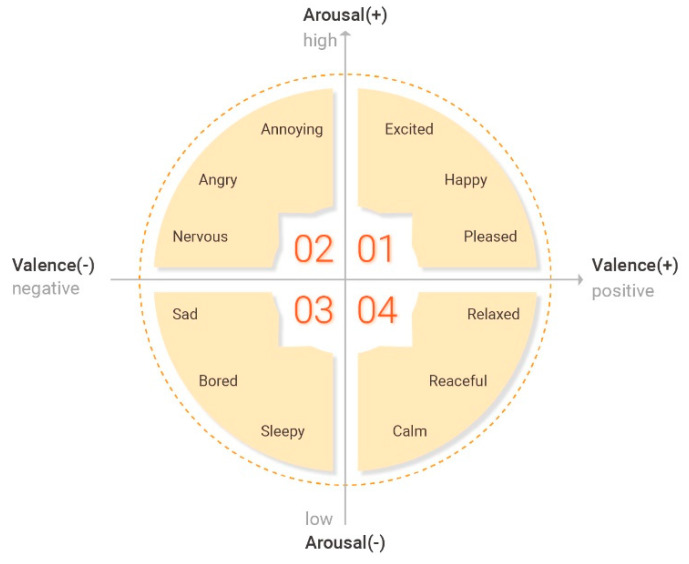
Thayer’s extended 2-dimensional emotion model.

Instead of using adjectives, one can select specific typical emotions, such as joy, surprise, fear, terror, anger, and sadness. The valence–arousal model has the advantage of depicting human emotional states in a continuous manner and allowing multiple emotions to be selected. However, it has a disadvantage in that there are vague emotions for which it is difficult to distinguish between the corresponding 2D index and broad emotion adjectives. When a typical emotion is selected, that emotion’s expression is clear and it is easy to classify speech according to that emotion. Therefore, this method is commonly used for representing emotions in the field of speech-based emotion recognition. Figure 2 presents an overview of the Thayer emotion model.

In this study, we used a typical emotion depiction method with clear representations of emotions. We focused on six typical emotions that are often used in the field of emotion recognition: neutral, happy, sad, angry, surprised, and bored.

#### 2.1.2. Speech Emotion Information

The speech signals vocalized by humans are acoustic signals that contain various types of information, such as linguistic and unique biometric information (speaker information). The objective of preprocessing is to extract the parameters that express the speech from speech signals such that they are useful for the next process based on the language and speaker information.

##### End-Point Detection

The speech of a speaker vocalized through a microphone includes slice and noise sections in addition to speech sections, which include language or speaker information. In the end-point extraction process, it is necessary to distinguish between the noise and speech sections from the input signals. The performance of the recognition system strongly depends on the accuracy of the end-point extraction, and generally, parameters such as the short-section algebraic energy and zero crossing rate are used. The algebraic energy is used to distinguish between the speech and noise sections, while zero crossing rate is used to distinguish between the speech interval and non-speech interval sound sections. In noiseless speech signals, the end-points can be accurately extracted to some degree using only the algebraic energy and zero crossing rate. However, if there is presence of noise, the end-point extraction becomes very difficult.

The short-section algebraic energy is the energy of a certain short section (frame). The end-points are extracted using a large energy change between the silent and speech sections based on the fact that the energy value of a silent section is lesser than that of a speech section. If the short-section energy is $E_{f}$, it is obtained by the following Equation (1).

(1) $$E_{f}=10\log\left(\sum_{n=0}^{N-1}x^{2}\left(n\right)\right)$$

where *N* is the total number of samples in one frame, and *x*(*n*) is the *n*th sample value of the input speech.

Similar to the algebraic energy, the zero crossing rate is calculated for each frame. It represents the number of times the speech signal input in one frame crosses the horizontal axis (zero point). This is used to distinguish between the speech interval and non-speech interval sound sections. The speech interval sound section has a large zero crossing rate because the energy is concentrated in a low-frequency band. Furthermore, the zero crossing rate in a silent section is modified by the surrounding environment. It is generally smaller than that of the non-speech interval sound and larger than that of the speech interval sound. When the zero crossing rate in each frame is *Z*, it is expressed as Equation (2).

(2) $$Z=\frac{1}{2}\left|sgn\left[x\left(t-n\right)\right]-sgn\left[x\left(t-n-1\right)\right]\right|,\;sgn\left[x\left(t\right)\right]=\{\begin{array}{c} 1,\;x\left(t\right)>0\\ -1,\;otherwise \end{array}$$

where *N* is the total number of samples in one frame, and *x*(*n*) is the *n*th sample value of the input speech.

##### Feature Parameter Extraction

The speech signals obtained by end-point extraction undergo the feature parameter extraction process. In this process, the feature parameters that express the features of the speech in each section (frame) of 10–30 ms are obtained. There are various feature parameters, such as energy, zero crossing rate, pitch cycle, formant, linear prediction coefficient cepstral, and the mel-frequency cepstral coefficient (MFCC). Studies comparing the performances of these feature parameters are in progress.

To perform emotion recognition based on speech information, it is necessary to select features that allow emotions to be distinguished, rather than the features that are typically used in speech recognition. Mel-frequency cepstral coefficients (MFCCs) are typical features that are used as parameters to represent the phonemes of speech, whereas pitch, energy, and pronunciation speed are prosody factors that are used in emotion recognition. Regarding speech parameters, the pitch and energy values that are calculated for defined intervals in a speech signal are used to calculate statistical information, such as average pitch, pitch standard deviation, maximum pitch, average energy, energy standard deviation, etc. This information can then be used for emotion recognition. Additionally, the Gaussian mixture model (GMM), hidden Markov model (HMM), support vector machine (SVM), and artificial neural network (ANN) algorithms are used in speech recognition and speaker recognition as identification methods during the pattern recognition stage [21,22].

As representative features of speech signals, we extracted pitch and energy, which contain prosodic features, as well as MFCCs, which contain phoneme features. We also calculated the delta values of each feature coefficient. In this study, the average, standard deviation, and maximum values of each feature coefficient were calculated and used as emotion recognition features to perform optimization via the “genetic algorithms as a feature selection method” (GAFS). Additionally, we used the SVM classifier to perform pattern recognition. Specifically, the accuracy of each kernel function in the SVM classifier was analyzed to identify features that can be used to classify and recognize each emotion accurately. A speech emotion database was constructed from the speech emotion information learned in this manner.

##### Preprocessing Process

The speech preprocess to extract the reliable feature vectors is composed of the division of speech signals in the frame unit, Hamming window, and end-point detection.

First, input speech signals are sampled at 16 kHz and used to extract features via the 16-bit pulse-code modulation method. A Wiener filter is then used to remove noise from the sampled speech signals.

We used the Hamming window when extracting pitches from the sampled speech signals [23,24]. Additionally, a Hamming window that overlaps neighboring frames by 50% is also applied. Next, end-point detection is performed to distinguish speech intervals and non-speech intervals in the speech signals and extract feature vectors from only the speech intervals. This prevents poor system performance based on invalid speech analysis and feature vector extraction during non-speech intervals.

##### Feature Extraction

To perform speech-based emotion recognition, it is necessary to identify how each emotion affects speech precisely. The emotions contained in speech are largely expressed through prosody information, such as pitch changes, energy changes, and pronunciation speed changes. To perform emotion recognition, it is necessary to identify the features in speech that accurately reflect this prosody information and perform appropriate modeling.

Regarding the correlation between prosody information and emotional speech, it is known from a statistical perspective that happy or angry speech generally has high energy and pitch with rapid pronunciation speed, whereas sad or bored speech generally has low energy and pitch with slow pronunciation speed [25,26]. The pitch and energy levels of speech can be modeled using statistical information, such as the average pitch and average energy of all pronounced speech intervals.

To create speech feature vectors, we extracted each frame unit’s pitch and energy, which include prosodic features, as well as MFCCs, which include phoneme features. We also calculated feature coefficient delta values. Ultimately, the average, standard deviation, and maximum values of each feature coefficient were calculated and feature vectors were created from these values.

We used the MFCC, a representative speech feature extraction method, to extract the speech features. The reason for using the MFCC as the feature vector is that the nonlinear mel unit is robust to noise because it reflects the human hearing characteristics well and can easily distinguish between information about basic frequencies. The MFCC was created based on the fact that the human hearing organs are sensitive to the low-frequency band but are relatively insensitive to the high-frequency band. It is a speech feature expressed by the mel scale. The mel scale, which was named by Stevens and others, expresses the relationship between the physical sound height and auditory perceived sound height.

In order to obtain the MFCC feature, the speech signals pass through an anti-aliasing filter and are then converted to digital signals *x*(*n*) through analog–digital conversion. The digital speech signals pass through a digital pre-emphasis filter that has high-pass filter characteristics.

This filter was used due to the following reasons. First, high-pass filtering is done to model the frequency characteristics of human outer/middle ears. This compensates for the attenuation of 20 dB/decade by radiation from the lips and only the vocal tract characteristics are obtained from the speech. Furthermore, it also compensates to some extent for the sensitivity of the hearing system to the spectrum range above 1 kHz. The characteristic of the pre-emphasis filter, *H*(*z*), is expressed as Equation (3), where a is in the range of 0.95–0.98.

(3) $$H\left(z\right)=1-az^{-1},\;0.9\leq a\leq 1.0$$

The pre-emphasis signals are covered by the Hamming window and are divided into frames of block units.

In the preprocess for extracting the speech feature vector, the speeches are processed in the frame units under the assumption that they are the normal sections for a duration of 10–30 ms. Generally, the frame size is 20–30 ms, and 10 ms is often used for the frame movement.

When speech signals are analyzed only in the time domain, it is difficult to sufficiently analyze the information contained in the signals. Hence, a technique for converting the signals of the time domain to the frequency domain is used for signal analysis. Among the methods used to express the power spectrum of the section signals, the MFCC, which is widely used for speech recognition, was selected to express the characteristics of the phonemes. Unlike the general cepstrum, the MFCC evenly divides the frequency bands at the mel scale. It can be used for emotion recognition because even the same phoneme can have a different form depending on the emotion contained in it.

In this study, the feature vectors were extracted by covering the signals with a 20-ms Hamming window and shifting them with 50% overlapping (10 ms). Speech signals are quasi-periodic signals composed of periodic and non-periodic signals. Therefore, the speech signals must be made more periodic by considering a periodic window. The formula for considering the window is Equation (4).

(4) $${\tilde{x}}_{l}\left(n\right)=x_{l}\left(n\right)\cdot w\left(n\right),\;0\leq n\leq N-1$$

The following Equation (5) expresses the Hamming window.

(5) $$w\left(n\right)=0.54-0.46\cos\left(\frac{2\pi n}{N-1}\right)$$

The speech signals of one frame were converted to the frequency domain using fast Fourier transform (FFT). The frequency band was divided into multiple filter banks and the energy of each bank was determined. The shape of the filter bank and the method of setting the center frequency were determined considering the frequency characteristics of the cochlea. A triangular shape filter was used, and the center frequency was located linearly until 1 kHz. Above this, it consisted of 20 banks distributed at the mel scale. Equation (6) is the mel-frequency linear transformation formula. To reduce the discontinuity of the border between the filters, the triangular filters were generally overlapped. The width of each filter bank was set from the center frequency of the previous filter bank to the center frequency of the next filter bank.

(6) $$mel=2595\;log10\left(1+\frac{f}{700}\right)$$

The final MFCC was obtained by performing an inverse discrete cosine transform (IDCT) after taking the log of the band energy. Regarding the MFCC coefficients, 12 coefficients from c1 to c12 were used. In addition, the frame log energy was used and the feature vector used as the input for speech recognition became the 13th vector. To reflect the characteristics of the modified values of the speech signals, derivatives called delta or SDC were added, and as a result, there were a total of 39 feature vectors using the MFCC.

The parameters used to extract the MFCC features are listed in Table 1.

**Figure 3:**
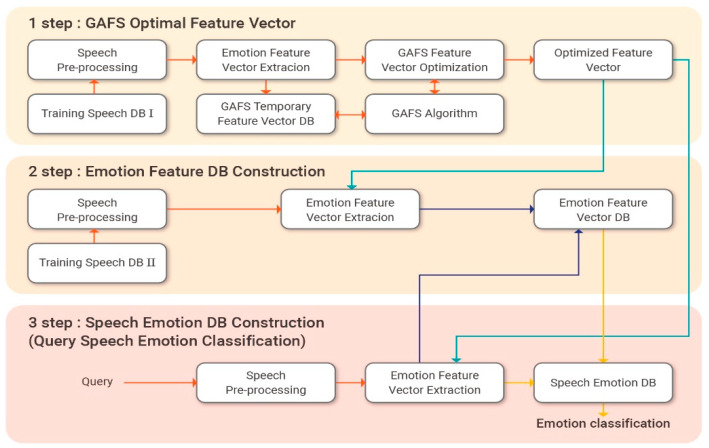
Speech signal feature vector extraction and emotion classification flow chart.

Figure 3 presents the workflow for the speech signal feature vector extraction and emotion classification process proposed in this paper.

Pitch is the height of the sound. The sound is high when the frequency of the vocal cords is large, and the sound is low when the frequency is small. The commonly used methods include the harmonic product spectrum, average magnitude difference function (*AMDF*), and the sub-harmonic to harmonic ratio. In this study, we adopted the *AMDF* method because it shows a high emotion recognition rate in a noisy environment.

Our pitch extraction process uses a 60-ms Hamming window to extract two to three pitches from each frame and passes speech signals through a low-pass filter with a blocking frequency of 800 Hz. Next, the average magnitude difference function (*AMDF*) is used to select the pitch with the minimum value among the extracted pitch candidates, as shown in Equation (7).

(7) $$AMDF_{n}\left(j\right)=\frac{1}{N}\sum_{i=1}^{N}\left|x_{n}\left(i+j\right)\right|,\;\;1\leq j\leq MAXLAG$$

Here, *N* is the number of samples and $x_{n}\left(i\right)$ is the *n*-th frame’s *i*-th sample value. MAXLAG denotes the maximum value of the pitch period that can be extracted.

The extracted pitch candidates are smoothed to prevent the pitch from changing rapidly between frames. If there is a short speechless frame interval (one to two frames) between speech intervals, it is processed as a speech interval with the average pitch value of the adjacent frames.

To calculate energy, we use the common log energy and Teager energy measures. Log energy is calculated as the log of the sum of the absolute values of the sample signal in a frame. Teager energy is a measure proposed by Kaiser. To calculate this measure, a filter bank is applied to a complex sinusoidal signal and the result is divided by a single frequency. The energy value is then calculated as shown in Equation (8) [27].

(8) $$TE_{n}\left(i\right)=\;f_{n}^{2}\left(i\right)-f_{n+1}\left(i\right)f_{n-1}\left(i\right),\;i=1\ldots FB$$

Here, $f_{n}\left(i\right)$ is the *n*th frame’s *i*-th filter bank coefficient and FB is the number of frequency bands. Teager energy signals have features that are robust against noise and speech signals are improved dynamically.

MFCCs include phoneme features and are widely used in the field of speech recognition. They can accurately represent speech characteristics at mel frequencies that are similar to the characteristics of human hearing.

The features of the speech were extracted in the frame unit. The mean, standard deviation, and interquartile range (IQR), which are statistical values, were calculated from the extracted baseline feature vector column and used as features for emotion classification.

##### Feature Vector Optimization Algorithm (GAFS)

We use the GAFS algorithm to optimize feature vectors. Feature selection is a nonlinear optimization problem. In the field of emotion detection, feature selection is a very difficult problem where one must find a feature set that satisfies the objective function, which represents emotion recognition performance improvement, among feature sets that stretch across dozens of dimensions. GAs were developed through studies on resolving this type of optimization problem and are known as domain-independent combination optimization methods. A GA can be applied wherever a function for obtaining an output is defined. A Ga begins with a population consisting of a set of individuals generated by a computer in a search space representing all possible solutions to the target function. It selects only suitable objects according to the objective function, which measures how well objects fit in their environment. Ultimately, the algorithm selects an optimal solution by repeating a process of evolution toward more optimized objects [28,29].

**Figure 4:**
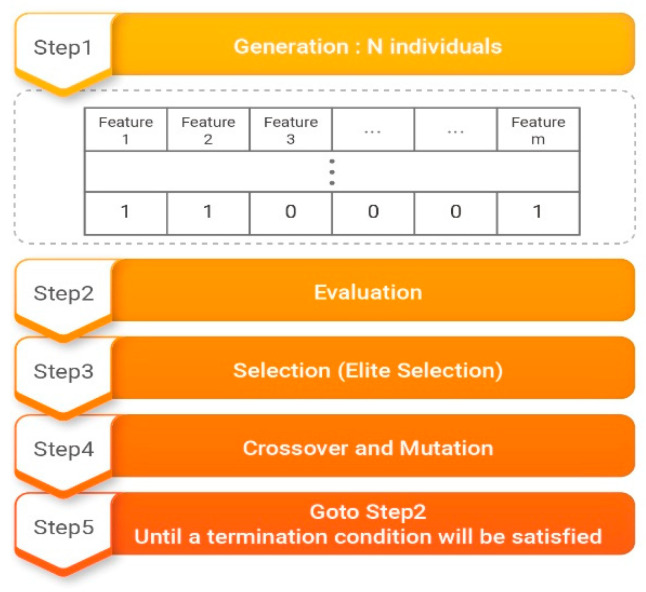
Genetic algorithms as a feature selection method (GAFS) algorithm.

We used the GAFS to optimize feature vectors, as shown in Figure 4. When a solution set is initially created, the length of the chromosomes is adjusted to match the quantity required by the objective function. Because the objective function consists of eight features, the length of the chromosomes is set to 10. The first stage consists of creating a predetermined population size N of objects with chromosome lengths of 10. In the second stage, the N objects are analyzed according to the objective function to determine their fitness. The fitness values found in this manner are used to select an elite population. Crossover and mutation are then applied according to preset crossover and mutation rates. Fitness is then rechecked, and steps two to five are repeated until the end conditions are satisfied.

##### SVM Classifier

The SVM classifier was used to recognize patterns in the emotion information contained in the optimized feature vectors. The SVM classifier finds an optimal hyperplane that minimizes the number of decision errors between two classes. Additionally, an SVM has a very simple structure compared to a neural network and has advantages in terms of generalization. This makes the SVM a popular choice in many application fields [30,31,32,33].

To classify the optimized feature vectors of the speech, the data access patterns of the SVM classifier are analyzed.

First, the discriminating equation that forms the basis of classification is defined as Equation (9).

(9) $$f\left(x\right)=\sum_{i=1}^{M}a_{i}^{*}z_{i}K\left(X_{i}^{*},\;X\right)+b^{*}$$

$X_{i}^{*}$ is the *i*th vector among the *M* support vectors obtained by learning. The optimization bias $b^{*}$ and the Lagrange multiplier $a^{*}$ are the solutions of the quadratic programming problem determined by learning. When the radial basis function (RBF) is used as the kernel function, $K\left(X_{i}^{*},X\right)$ can be expressed as Equation (10).

(10) $$K\left(X_{i}^{*},X\right)=exp\left(-\frac{{||X_{i}^{*}-X||}^{2}}{\sigma^{2}}\right)$$

**Figure 5:**
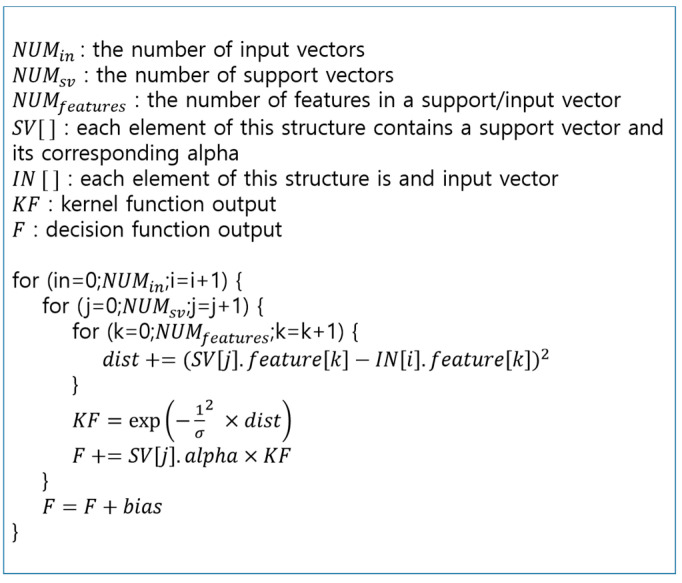
Pseudocode for support vector machine (SVM)-based classifier.

σ is a parameter related to the width of the RBF. The reason for using the RBF as the kernel function is that it is the preferred kernel function when the linear classification of the input signals is impossible. The pseudocode corresponding to the discriminating equations composed of Equations (9) and (10) is shown in Figure 5.

$NUM_{in},\;NUM_{sv}$, and $NUM_{feature}$ represent the number of input vectors, the number of support vectors, and the dimensions of the support and input vectors. SV is a structure that stores the support vector and is composed of a support vector with a dimension of $NUM_{feature}$ and the corresponding Lagrange multiplier. *IN* is a structure that stores the input vector. The vector in each structure is stored as an array, and its name is the feature. Dist is a value corresponding to ${||X_{i}^{*}-Z||}^{2}$ in Equation (10), *KF* is the resulting value of the kernel function expressed as Equation (10), and *F* is the *f*(*x*) on the left side of Equation (9).

The pseudocode has three loops. The first loop substitutes the input vectors in the discriminating equation sequentially. The second loop matches each input vector to every support vector one by one. The last loop performs the vector operation of one input vector and one support vector. The support vectors in this study are sequentially loaded for each input vector and used for calculation in units of the vector elements. In other words, the first element of the first support vector to the last element of the last support vector are read in turn for one input vector, and they are also read in the same sequence for the next input vector.

The kernel functions that are generally used in SVMs include linear kernels, Gaussian radial basis function (RBF) kernels, polynomial kernels, and sigmoid kernels, as shown in Table 2 [34,35]. We tested linear, Gaussian RBF, polynomial, and sigmoid kernels to classify emotions and perform recognition.

#### 2.1.3. Image Emotion Information

To extract image-based emotion information, we used RGB values. To measure emotion scales, 20 color emotion models from Hewlett-Packard (HP)’s “The Meaning of Color” were selected as typical elements. A five-point-scale survey was used to perform factor analysis and correspondence analysis, and emotion spaces were created for each color.

RGB values were extracted at certain points in each image and each RGB value was saved in a database. To understand degrees of emotion according to color distributions, the RGB values of the color models were interpreted as 3D x, y, and z coordinates. The distances between the extracted colors were calculated and included in the shortest-distance color model. Each image’s color model distribution was saved in the 20 color model fields of the database.

Each of the 20 selected color models measured the distance to the RGB of each pixel extracted from the image in three-dimensional space. If the RGB for one pixel of the extracted image was (0, 0, 0), its distance to each color model belonged to the color model “Navy”, as shown in Table 3.

The analyzed color images were then transformed into color measurement values and visualized as a graph. Emotion words were matched according to the highest corresponding measurement values [36,37].

The emotional vocabulary for the color information was selected in four steps. In step 1, the adjectives were included in the meaning-based English dictionary WordNet. In step 2, 662 emotional words were extracted by comparing the results of a survey with the vocabulary in step 1. In step 3, words having duplicate meanings and low frequency were removed. As a result, the vocabulary was compressed to 341 words. In the last step, a total of 26 emotional words representing the colors were extracted by selecting the representative words and antonyms. Table 4 lists the extracted emotional words based on the color information.

After converting the emotion elements and emotion words into a 2D space, this new space was used to obtain the coordinates of the emotion words and emotion elements. These coordinates were used to measure distance and the resulting distances were considered to represent the relationships between emotion elements correlated with emotion words. A smaller distance between coordinates indicates that the corresponding relationship is more significant (inversely proportional). A larger color distribution in an image indicates that the contained relationships are more significant (directly proportional). The inverse of distance was calculated to measure distance ratios, as shown in Equation (11).

(11) $$D_{ik}=\frac{d_{ik}^{-1}}{\sum_{j=1}^{20}d_{ij}^{-1}}$$

This equation yields the distance ratio of the emotion word *i* relative to the emotion element *k*. The numerator represents the distance between the actual emotion word *i* and emotion element *k*. The denominator represents the sum of the inverses of the distances between the emotion word *i* and the 20 emotion models.

Table 5 and Table 6 show the coordinates of each emotional word and the color emotion element measured in the color–emotion space.

#### 2.1.4. Music Emotion Information

The proposed system can use music information and each user’s emotion history to recommend music that is appropriate for the user’s current mood according to their speech emotion information. Euclidean distance was used as a measure of similarity between audio sources and music emotion information [38,39].

The proposed algorithm extracts information regarding the type of music that was most often played during the emotional state that is most similar to a user’s current emotion to find the most emotionally relevant music.

(12) $$Score_{i}=\sum_{e=1}^{6}\frac{uEmotion_{e}}{100}\times hEmotion_{i,\;e}$$

Equation (12) is used to calculate scores for the songs that a user has listened to based on that user’s current state (i.e., $uEmotion_{e}$). Here, i is a song in the music emotion database and e is the emotion information for the *i*-th song in the music emotion database. Therefore, Equation (12) can be used to set the priority of all songs in the music emotion database.

To collect a music list up to rank x, emotion information standardization is performed by using Equation (13) to calculate Euclidean distance.

(13) $$nEmotion_{i,k}=\frac{Emotion_{i,\;k}}{\sum_{m=1}^{8}Emotion_{i,\;m}}$$

Here, $Emotion_{i,k}$ is the value that corresponds to the *i*-th song’s *k*-th emotion category. The proposed algorithm calculates Euclidean distances and generates a recommendation list sorted in ascending order of distance.

(14) $$\sqrt{\sum_{m=1}^{8}{\left(nEmotion_{1st,\;m}-nEmotion_{i,\;m}\right)}^{2}}$$

Equation (14) calculates the similarity between songs based on the song with the highest value according to Equation (12) and the standardized emotion information. Here, *i* represents all of the songs in the music emotion database.

### 2.2. Emotion Collaboration Filtering Moule

**Figure 6:**
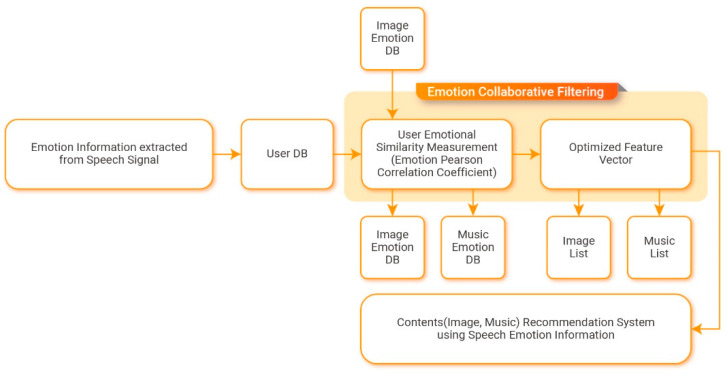
Emotion collaborative filtering module configuration.

Collaborative filtering is a method that predicts preferences regarding items by collecting the preferences of other users with similar tastes. It begins with the assumption that there are general trends and patterns in tastes and that people will maintain their past tastes in the future. Based on the principle that the preferred items vary depending on user emotions, content is recommended according to user emotions by incorporating collaborative filtering and static emotion information received from users in real time. Figure 6 presents the structure of the emotion collaborative filtering module.

Pearson’s correlation coefficients are calculated using the evaluation values for items that were evaluated by two users. This allows the proposed algorithm to detect similarities between pairs of users. By including the emotion information calculated previously, the Pearson correlation algorithm performs clustering using dynamic emotion information received from users in real time and user personal information. The evaluation scores of the created user groups are then used to measure the similarities between users regarding content according to user emotions. The measured degrees of similarity have values between −1 and 1. As the value approaches one, users are considered to be more similar. As the value approaches −1, users are considered to be more dissimilar. When the level of similarity is zero, it indicates that users have no correlation. Equation (15) is used to calculate the emotional Pearson correlation coefficients incorporating emotion information.

(15) $$w_{a,u,e}=\sum_{i=1}^{m}\frac{\left(r_{a,i,e}-{\bar{r}}_{a,e}\right)*\left(r_{u,i,e}-{\bar{r}}_{u,e}\right)}{\sqrt{\sum_{i=1}^{m}{\left(r_{a,i,e}-{\bar{r}}_{a,e}\right)}^{2}}\sqrt{\sum_{i=1}^{m}{\left(r_{u,i,e}-{\bar{r}}_{u,e}\right)}^{2}}}$$

Here, $w_{a,u,e}$ is the similarity between a user and a neighboring user, *a* is the target user, *u* is the neighboring user, *e* is the emotion, m is the number of items evaluated by both a and *u*, $r_{a,i,e}$ is the evaluation score of user a for item *i* when considering *e*, $r_{u,i,e}$ is the evaluation score of user *u* for item *i* when considering *e*, ${\bar{r}}_{a,e}$ is the overall evaluation score of user *a* for *e*, and ${\bar{r}}_{u,e}$ is the overall evaluation score of user *u* for *e*. The formula in the denominator refers to the standard deviation of user a for e and the standard deviation of user *u* for *e*.

After performing clustering based on the measured levels of similarity between users and user personal information, the evaluation data from the created groups of users are used to predict preferences (i.e., evaluation scores) for items that the users have not seen. By using evaluation scores that were directly provided by the users and emotions that correspond to the current circumstances, it is possible to recommend personalized content. Equation (16) defines the prediction algorithm for evaluating scores by considering emotion information.

(16) $$P_{a,i,e}={\bar{r}}_{a,e}+\frac{\sum_{u=1}^{n}w_{a,u}*\left(r_{a,i,e}-{\bar{r}}_{u,e}\right)}{\sum_{u=1}^{n}w_{a,u}}$$

Here, $p_{a,i,e}$ is the predicted evaluation score for item *I*, a is the target user, u is the neighboring user, e is the emotion, n is the number of neighboring users with evaluation scores, ${\bar{r}}_{a,e}$ is the overall average evaluation score of user a for e, ${\bar{r}}_{u,e}$ is the overall average evaluation score of user *u* for *e*, and $r_{u,i,e}$ is the evaluation score of user *u* for item *i* considering *e*. Finally, $w_{a,u}$ is the level of similarity between users in terms of the emotional Pearson correlation coefficients (i.e., the level of similarity between *a* and *u*).

### 2.3. Mobile Applcation

A user can use a content recommendation mobile application that employs emotion information and emotion preferences to register their personal information. This registered personal information is then stored in the user database. Users who have stored their personal information can measure their own speech signals using the application. The GAFS and SVM algorithm are used to convert the measured speech signals into six types of standardized emotion information values in the emotion classification module. The emotion collaborative filtering module is used to find the level of similarity between a user’s emotion information values for each content item and the values of other users so that the system can recommend content that elicited specific emotions in other users with similar preferences. Then, a recommendation list is provided according to the content emotion information values that match the standardized emotion information values. The content provided in the recommendations is stored in the image emotion database and music emotion database. The recommended content, emotion measurement values, recommendation list, and measurement graphs are presented to the user by a content recommendation mobile application interface.

## 3. System Implementation Results and Performance Evaluation

To consider user speech emotion information and recommend matching content, the proposed system defines human emotions according to six categories: neutral, happy, sad, angry, surprised, and bored. The GAFS and SVM algorithm are used to classify normalized speech into speech emotion information. Additionally, content (images and music) is classified into content emotion information using factor analysis, correspondence analysis, and Euclidean distance. Finally, emotional preferences are predicted using collaborative filtering. The proposed system is designed to match emotion information and emotional preferences extracted in this manner. This allows it to recommend content that matches a user’s current emotions.

First, the GAFS and SVM algorithm are used to perform extraction and analysis on the features of speech data that are recorded using a smartphone microphone. Next, the data are stored in the emotion speech database according to the six defined standardization types (neutral, happy, sad, angry, surprised, and bored). In the image emotion database, emotion colors and emotion words are arranged on the same 2D spatial plane and the distances between emotion colors and emotion words are measured to identify related information. Factor analysis is then applied to the measured information to perform verification and extract typical emotion words. These data are stored in the image emotion database according to the six defined types. The music emotion database allows Euclidean distance to be used to recommend music that is appropriate for a user’s current emotions based on music information and user emotion information.

In this study, we constructed a Korean emotional speech database, appropriate for the Korean language, using the experimental data for the recognition of personalized speech emotions. The language was Korean and the speech data were composed of the speech of 30 persons (15 males and females each) in their 20s and 30s who were ordinary people, not speech actors. Apart from “user registration”, there were six items (user registration, greetings, commands, emotions, living information, and dates/times). The speech files of five items (50 words and sentences) were used, excluding the user registration. The number of speech data per person was 300, and the database contained 9000 speech data in total.

The microphone height was set to 74 cm, the distance to the speaker was 2 m, and the distance between two microphones was 20 cm. The two microphones were experimented with at five angles. The two people spoke at 0°, −30°, −15°, +15°, +30° from the center of the two microphones. This study used only the speech data where the angle difference between the two microphones was 0°. The speeches were recorded in a quiet environment. The data were stored at the sampling rate of 16 kHz, as 16-bit data, in linear pulse code modulation (PCM) format. The silent section was set as 300 ms.

When the speech data were recorded, the actual situation was reflected. The monitor agent checked the incorrect vocalizations by the speakers and asked them to speak the words again.

1. The speaker information document was prepared before recording.
2. The precautions and emotional state during recording were explained to the speaker and the speaker practiced the vocalization of the words and sentences for approximately five minutes before recording.
3. Each speaker vocalized 50 times with one emotion, 10 times each, at five positions.
4. The user registration item was recorded only with the neutral emotion.
5. The monitor agent checked the incorrect vocalizations made by the speakers during recording and asked them to speak the words again.
6. In the case of re-vocalization, the vocalization of the speaker may be unnatural if it is corrected excessively. Therefore, if the speaker made incorrect vocalization even after several re-vocalizations, their vocalization was not corrected excessively, and they were allowed a resting time before re-vocalization to encourage natural vocalization.

As shown in Table 7, the emotional speech database consists of five items that represent the user registration, greetings, living information, commands, and emotions.

The 9000 emotional speech data in the database were evaluated on a five-point scale by a group of seven emotion evaluators regarding how well the speech expressed the emotions. A total of 2400 good-quality data that expressed the emotions well with no background noise were selected, with a 1:1 ratio of male and female data.

The evaluation of emotions was performed according to the standard chart as shown in Table 8. The final speech database was selected separately for male and female speech data. A total of 400 data were selected, with 200 male and female data each for each emotion. Table 9 shows the standard scores for the database selection and the mean scores of the final speech data.

The emotions of the experimental data were categorized as neutral, happy, sad, angry, surprised, and bored, which are the emotion categories of the emotional speech database. For each emotion, 400 data were selected as the experiment data, and the data were analyzed with a window size of 250, time step of 78, and a frame of 15 ms unit.

The collected data were divided into learning-stage data and recognition-stage data. The classification accuracy of the recognition stage’s features was calculated and verified through classification and comparison using the GAFS and SVM algorithm, which were trained during the learning stage. If the learning data account for less than 10% of the total data, accuracy is very low. Therefore, a sufficient amount of learning data must be provided. When the learning data ratio is 50%, the accuracy reaches 0.975. However, although accuracy generally increases as the amount of learning data increases, accuracy tends to decrease as the learning data ratio approaches 100%. Therefore, the ratio of learning data to recognition data was set to 50:50. The trained model and newly entered recognition data were used to calculate emotion recognition accuracy rates and the feasibility of the trained model was thoroughly reviewed.

In this study, performance was evaluated using precision, recall, and F-measure values, which are the main performance analysis metrics used in automatic classification and machine learning evaluations, to select an optimal SVM kernel function. In most cases, precision and recall can be calculated using a 2 × 2 contingency table for each category [40,41]. Table 10 compares the ground truth classification results to the recognition system classification results.

In Table 10, *a* denotes the number of data that are correctly classified for a particular emotion category, *b* denotes the number of data that are incorrectly classified for a particular emotion category, *c* denotes the number of data that should be classified as a certain emotion category but are incorrectly classified, and *d* denotes the number of data that do not actually belong in a particular emotion category and that the system cannot find. Equations (17)–(19) are used to calculate precision, recall, and *F-measure* values, respectively.

(17) $$Precision\;\left(P\right)=\;\frac{a}{a+b}$$

(18) $$Recall\;\left(R\right)=\;\frac{a}{a+c}$$

(19) $$F-measure\;\left(F\right)=\;\frac{2RP}{R+P}$$

The speech emotions contained in the speech emotion database were extracted from 50 types of speech performed by users. To choose a kernel function suitable for the target user emotions, emotion classification accuracy was verified by testing different SVM kernel functions, as shown in Table 11. As shown in the results in Table 11, the RBF kernel yields a recognition accuracy of 86.98%, making it the best-performing kernel function. The lowest recognition accuracy (77.74%) can be observed for the sigmoid kernel function.

The kernel function recognition results for each specific emotion are discussed below.

Table 12 lists the linear kernel function classification results for each emotion. The average accuracy is 83.99%. The recall for each emotion is as follows: neutral (82.19%), happy (86.33%), sad (86.87%), angry (82.37%), surprised (82.67%), and bored (83.50%).

The polynomial kernel function yields an accuracy of 86.17%. The recall for each emotion is as follows: neutral (85.89%), happy (86.26%), sad (87.61%), angry (86.47%), surprised (85.43%), and bored (85.34%). These recall results are shown in Table 13.

The RBF kernel yields an emotion classification accuracy of 94.77%. The recall for each emotion is as follows: neutral (90.84%), happy (100%), sad (95.83%), angry (97.50%), surprised (93.22%), and bored (91.20%). Table 14 lists the RBF kernel results.

The sigmoid kernel function yields an emotion classification accuracy of 82.97%. The recall for emotion is as follows: neutral (81.80%), happy (82.60%), sad (86.66%), angry (81.64%), surprised (82.04%), and bored (83.10%). Table 15 lists these results.

Table 16 lists the precision, recall, and F-measure results, which are performance evaluation metrics for each kernel function. The nonlinear SVM RBF kernel exhibits the best performance in terms of precision (94.70%), recall (94.77%), and F-measure (94.71%). The sigmoid kernel function exhibits the worst performance in terms of precision (84.00%), recall (84.87%), and F-measure (82.82%).

Furthermore, when the accuracy rate, reproduction rate, and the F-measure, which are the performance measures, were analyzed using the RBF kernel function, the accuracy rate was 94.70%, the reproduction rate was 94.77%, and the F-measure was 94.71%.

To evaluate the performance of the recommendation system proposed in this paper, we adopted the mean absolute error (MAE) metric. To determine the accuracy of the recommendation system, predicted preferences and actual preferences were measured and compared for each item. The results indicate how similar the predicted evaluation scores and actual evaluation scores are on average. The dataset used in our experiments contained content data (images and music) for each emotion generated from the speech emotion information.

**Figure 7:**
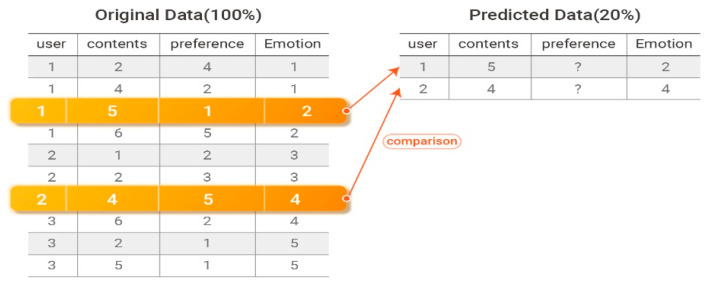
Removal of 20% of the data from the original data.

Experiments were performed by randomly selecting 80% of the dataset for training and predicting the remaining 20%, as shown in Figure 7.

**Figure 8:**
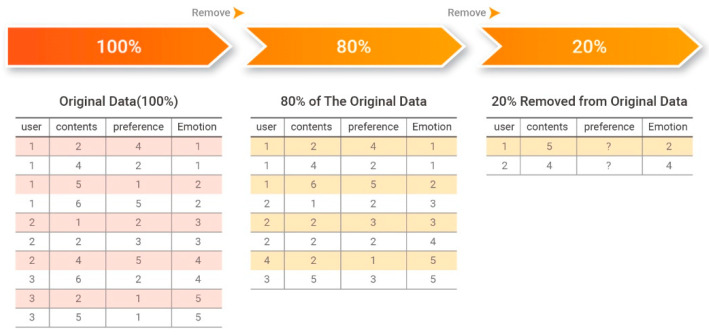
Comparison of original and predicted affinity data.

The performance of the proposed system was evaluated by comparing the predicted 20% of the data to the 20% of the original data that were withheld for training. Figure 8 presents an example of comparing predicted preference data to withheld preference data.

MAE is the average of the absolute errors between two groups of values that are comparison targets. It is an index that represents how similar predicted evaluation scores are to actual user evaluation scores on average. The performance of the recommendation system is considered to be better when the MAE value is smaller. An MAE value of zero indicates that the recommendation system is perfectly accurate. Equation (20) defines the MAE calculation.

(20) $$\mathrm{MAE}=\;\frac{\sum_{i=1}^{n}\left|p_{i}-q_{i}\right|}{n}$$

Here, $p_{i}$ is the actual preference of user p, $q_{i}$ is the predicted preference of user q, and n is the number of content items used by user p.

In this study, the MAE values were normalized to a range of zero to one and inverted such that zero indicates that none of the values match and one indicates that all of the values match. In Equation (21), the normalization formula is included in the MAE calculation.

(21) $$\mathrm{MAE}=1-\frac{1}{n}\sum_{i=1}^{n}(\frac{\left|p_{i}-q_{i}\right|}{MAX-MIN})$$

Here, $p_{i}$ is the actual preference of user p, $q_{i}$ is the predicted preference of user q, n is the number of content items used by user p, MAX is the maximum value of $p_{i}-q_{i}$, and MIN is the minimum value of $p_{i}-q_{i}$.

Table 17 presents the results of evaluating the proposed recommendation system’s performance for each emotion according to the normalized MAE value. The performance evaluation results reveal an average accuracy of 87.43%, indicating that the recommendation system’s performance is good.

**Figure 9:**
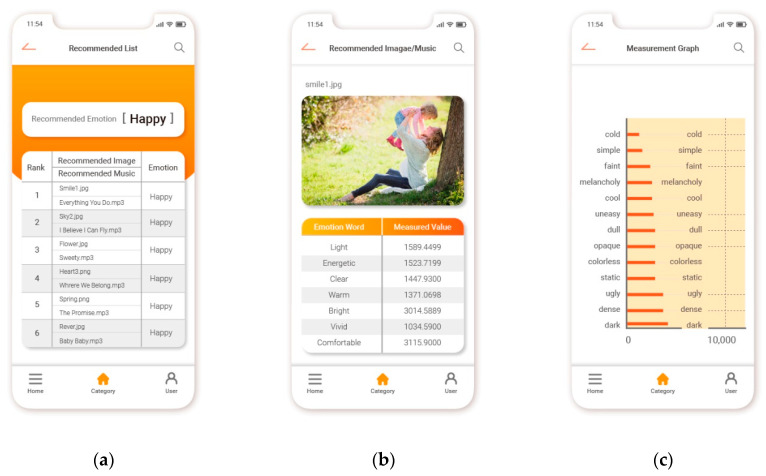
The results achieved by the proposed system: (**a**) presents the recommended emotion content and measurement values, (**b**) presents the emotion content recommendation list, and (**c**) presents a measurement graph of emotion content.

In this study, a content recommendation system that uses individual speech emotion information and collaborative filtering was implemented as a mobile application. Recognized emotion information values were used to predict user preferences and recommend content. Figure 9 presents the results achieved by the proposed system. Specifically, Figure 9a presents the recommended emotion content and measurement values, Figure 9b presents the emotion content recommendation list, and Figure 9c presents a measurement graph of emotion content.

The system implemented in this study uses user speech emotion information and collaborative filtering to match content emotion information with user emotion information that is standardized into six categories. Next, the proposed system searches for preferred content (images and music) according to user selection patterns in a standardized database and recommends content in a ranked order. Optimal content can be recommended to users by displaying an emotional analysis chart representing the analyzed content.

## 4. Conclusions

User speech emotion information and collaborative filtering were used to standardize content emotion information into six categories (neutral, happy, sad, angry, surprised, and bored). The GAFS, SVM algorithm, factor analysis, correspondence analysis, and Euclidean distance were used to acquire and recognize speech and content (images and music) emotion information and extract reliable data. Precision, recall, and F-measure performance indicators were analyzed for each kernel function of the SVM algorithm. The results revealed that the RBF kernel yielded the best performance, with a precision of 94.70%, recall of 94.77%, and F-measure of 94.71%. Additionally, collaborative filtering was used to predict user emotion preferences and the performance of the recommendation system was evaluated for each emotion according to MAE, which was normalized to increase accuracy. The results revealed an average accuracy of 87.2%, which indicates that the proposed recommendation system’s performance is good. A service that recommends content (images and music) according to user speech emotions was implemented based on the acquired emotion information and used to recommend content matching each individual. The recommendation method proposed in this paper has high emotion recognition precision and recall compared to existing methods and has a very simple structure because it uses existing machine learning algorithms. In the future, the proposed method may be extended to human-oriented applications in a variety of environments, such as emotional interactions that occur between people based on human emotions. It may also be used effectively in intelligent systems that recognize emotional exchanges during interactions between humans and machines. The proposed system is helpful for considering user characteristics and increasing user satisfaction by recommending content matching user emotions.

In future studies, it will be necessary to analyze and study various algorithms for increasing recognition rates, as indicated by the speech emotion recognition results presented in this paper. Therefore, emotions that are extracted from facial expressions and speech will be used to implement systems with more stable recognition rates. Additionally, it will be necessary to collect various biometric data and analyze their characteristics to determine whether it is possible to judge emotions based on a small number of objective features. Data should be collected using a more sophisticated experimental design than that used in this study and research should focus on the direction of selecting models and features based on data that can reduce individual differences. Additionally, various machine learning algorithms other than the SVM should be considered.

## Figures and Tables

**Table 1:** Feature extraction parameters.

Sampling Rate	16 kHz, 16 bit
Pre-Emphasis	0.97
Window Type	Hamming Window
Window Size	20 ms
Shift Size Feature Parameters	10 ms MFCC + Energy (+Δ+ΔΔ/+SDC)

**Table 2:** The typical kernel functions applied to SVM classifier.

Kernel Function	Equation
Linear Function	K(x, y)=(x·y)
Gaussian Radial Basis Function	$K\left(x,\;y\right)=exp\left(\frac{{-||x-y||}^{2}}{\sigma^{2}}\right)$
Polynomial Function	$K\left(x,\;y\right)={\left(x\cdot y\right)}^{degree}$
Sigmoid Function	K(x, y)=thah(k(x⋅y)−θ)

**Table 3:** Distance of RGB color value in color space.

No	Color Name	RGB Value			Distance
		R	G	B	
1	Bright Red	255	35	40	260.5
2	Blue	0	93	199	219.7
3	Brown	96	47	25	109.8
4	Bright Yellow	255	214	10	333.0
5	Orange	255	91	24	271.8
6	Purple	140	43	137	200.5
7	Beige	232	203	173	353.5
8	Lime	94	168	34	195.4
9	Lavender	130	101	182	245.4
10	Olive Green	84	82	28	120.7
11	Burgundy	127	37	36	137.1
12	Green	0	130	63	144.5
13	Light Pink	251	188	172	357.7
14	Fuchsia	245	119	158	314.9
15	Light Blue	128	192	217	316.8
16	Navy	0	38	100	107.0
17	Greenish Yellow	199	181	0	269.0
18	Terracotta	172	165	26	239.8
19	Teal Blue	0	177	162	239.9
20	Neutral Gray	128	128	128	211.7

**Table 4:** Color emotion words.

Bright	Warm	Clear
Energetic	Pretty	Strong
Comfortable	Luxurious	Light
Vivid	Dynamic	Melancholy
Hot	Dark	Cool
Opaque	Dull	Ugly
Faint	Uneasy	Simple
Dense	Colorless	Static
Cheerful	Cold	

**Table 5:** Color coordinates of color–emotion space.

Index	Color Name	X	Y
C1	Bright Red	−0.670	0.746
C2	Blue	−0.639	−0.073
C3	Brown	0.557	0.202
C4	Bright Yellow	−0.836	0.041
C5	Orange	−0.659	0.359
C6	Purple	0.192	0.317
C7	Beige	0.131	−0.526
C8	Lime	−0.225	−0.152
C9	Lavender	−0.141	−0.137
C10	Olive Green	0.728	0.156
C11	Burgundy	0.548	0.299
C12	Green	0.213	0.021
C13	Light Pink	0.021	−0.560
C14	Fuchsia	−0.276	−0.053
C15	Light Blue	−0.460	−0.552
C16	Navy	0.441	0.211
C17	Greenish Yellow	0.133	−0.104
C18	Terracotta	0.322	0.197
C19	Teal Blue	0.011	−0.204
C20	Neutral Gray	0.609	−0.188

**Table 6:** Emotion coordinates of color–emotion space.

Index	Emotional Name	X	Y
1	Bright	−0.598	−0.264
2	Clear	−0.336	0.284
3	Warm	−0.322	0.228
4	Pretty	−0.488	−0.100
5	Energetic	−0.489	0.294
6	Comfortable	−0.247	−0.245
7	Strong	−0.274	0.341
8	Light	−0.233	−0.705
9	Luxurious	−0.240	−0.050
10	Dynamic	−0.471	−0.020
11	Vivid	−0.461	−0.013
12	Hot	0.278	0.347
13	Melancholy	0.473	0.070
14	Cool	0.475	−0.337
15	Dark	0.946	0.389
16	Dull	0.555	−0.333
17	Opaque	0.529	−0.447
18	Faint	0.385	−0.478
19	Ugly	0.692	0.142
20	Simple	0.321	0.067
21	Uneasy	0.367	0.364
22	Colorless	0.599	0.017
23	Dense	0.254	0.767
24	Static	0.631	0.027
25	Cold	0.282	−0.353
26	Cheerful	−0.612	−0.091

**Table 7:** The emotional speech database consists of five items.

Item	Words and Sentences
User Registration	(1) User Registration. (2) My name is ○○.
Greetings	(1) Hi. (2) How are you? (3) I miss you. (4) Long time no see! (5) What did you play with? (5) Bye. (6) I’ll be back. (7) See you later. (8) Come on. (9) Where are you going?
Living Information	(1) Tell me the weather today. (2) Is it raining? (3) Is it cool? (4) What’s the temperature?
Commands	(1) Stop. (2) Go up. (3) Go down. (4) Go back. (5) Go to the left. (6) Go to the right (7) Go forward. (8) Turn. (9) Don’t. (10) Stop it. (11) No. (12) Get up. (13) Sit down. (14) Do whatever you want. (15) Bring it here. (16) Come here. (17) Go that way. (18) Go this way.
Emotions	(1) Do something pretty. (2) Give me a wink. (3) Dance for me. (4) Good boy. (5) Are you okay? (6) Good. (7) Well done. (8) I couldn’t. (9) I love you. (10) You are pretty. (11) Will you be scolded? (12) Are you sick? (13) One more time. (14) Be quiet.
Data/Time	(1) What day is it today? (2) What time is it now?

**Table 8:** The emotion evaluation standard chart.

Evaluation Score	Evaluation Standard
1	Not this emotion at all.
2	The emotion is very weak.
3	It contains that emotion. (Normal)
4	Emotion is good.
5	The emotion is correct. (Excellent)

**Table 9:** The standard scores for the database selection and the mean scores of the final speech data.

	Male Database	Female Database	Average Score
Neutral	4.57	4.17	4.78
Happy	4.00	4.14	4.33
Sad	3.85	3.71	4.15
Anger	4.42	4.14	4.59
Surprised	4.00	4.14	4.35
Bored	3.82	3.85	4.23

**Table 10:** 2×2 contingency table for performance analysis of recognition method.

	Appropriate Emotion	Inappropriate Emotion
System Search	*a*	*b*
System Not Search	*c*	*d*

**Table 11:** Emotion recognition accuracy according to kernel function (%).

Classification		Neutral	Happy	Sad	Angry	Surprised	Bored	Average
Training	Linear	88.03	81.00	77.06	80.22	77.84	83.41	81.26
	Polynomial	88.67	86.84	88.19	88.97	88.97	88.77	88.40
	Gaussian Radial Basis Function (RBF)	92.97	90.63	92.19	92.97	89.06	92.19	91.67
	Sigmoid	86.26	81.09	82.66	77.97	65.47	80.94	79.07
Test	Linear	87.19	74.69	78.59	75.47	73.91	81.25	78.52
	Polynomial	85.78	80.94	86.41	87.03	80.94	79.38	83.41
	Gaussian Radial Basis Function (RBF)	89.06	92.19	86.72	87.50	81.25	85.16	86.98
	Sigmoid	83.28	74.69	85.63	73.91	73.91	75.00	77.74

**Table 12:** Linear kernel function-based emotion recognition results (%).

		Neutral	Happy	Sad	Angry	Surprised	Bored	Total
Linear Kernel Function	Neutral	82.19	2.86	3.81	4.48	3.90	2.76	100.00
	Happy	1.05	86.33	2.10	1.05	6.31	3.16	100.00
	Sad	1.88	3.13	86.87	2.50	3.75	4.75	100.00
	Angry	5.42	3.73	1.36	82.37	2.37	4.75	100.00
	Surprised	2.22	4.89	4.44	2.67	82.67	3.11	100.00
	Bored	3.50	4.00	3.00	2.67	3.33	83.50	100.00

**Table 13:** Polynomial kernel function-based emotion recognition results (%).

		Neutral	Happy	Sad	Angry	Surprised	Bored	Total
Polynomial Kernel Function	Neutral	85.89	2.39	2.62	3.24	3.78	2.08	100
	Happy	2.75	86.26	2.23	3.09	1.55	4.12	100
	Sad	2.23	2.24	87.61	2.23	3.25	2.44	100
	Angry	2.21	2.21	3.77	86.47	3.77	1.57	100
	Surprised	2.55	3.91	2.25	1.35	85.43	4.51	100
	Bored	2.66	2.84	2.84	3.55	2.77	85.34	100

**Table 14:** RBF kernel function-based emotion recognition results (%).

		Neutral	Happy	Sad	Angry	Surprised	Bored	Total
RBF Kernel Function	Neutral	90.84	0.00	1.53	0.76	1.53	5.34	100.00
	Happy	0.00	100.00	0.00	0.00	0.00	0.00	100.00
	Sad	0.83	0.00	95.83	1.67	1.67	0.00	100.00
	Angry	0.00	0.00	0.00	97.50	2.50	0.00	100.00
	Surprised	0.00	0.00	4.24	1.69	93.22	0.85	100.00
	Bored	2.40	0.00	2.40	0.80	3.20	91.20	100.00

**Table 15:** Sigmoid kernel function-based emotion recognition results (%).

		Neutral	Happy	Sad	Angry	Surprised	Bored	Total
RBF Kernel Function	Neutral	81.80	3.10	3.90	4.50	3.80	2.90	100.00
	Happy	2.59	82.60	2.22	2.96	4.82	4.81	100.00
	Sad	1.67	1.66	86.66	3.00	3.67	3.34	100.00
	Angry	5.25	1.97	2.95	81.64	2.94	5.25	100.00
	Surprised	2.86	6.53	2.04	4.49	82.04	2.04	100.00
	Bored	3.94	4.41	2.82	2.44	3.29	83.10	100.00

**Table 16:** Precision, recall, and F-measure by kernel function.

	Precision	Recall	F-Measure
Linear	86.23	85.93	83.44
Polynomial	87.00	87.46	84.82
RBF	94.70	94.77	94.71
Sigmoid	84.00	84.87	82.82

**Table 17:** Performance evaluation of recommendation system by emotion using mean absolute error (MAE) algorithm.

Emotion	Accuracy (Unit: %)
Neutral	83.6
Happy	92.5
Sad	87.9
Angry	89.8
Surprised	86.5
Bored	84.3
Total	87.43

## Data Availability

The data presented in this study are available on request from the corresponding author.
